# Functional stability assessment and adaptation for critical infrastructure facilities

**DOI:** 10.3389/frai.2026.1777258

**Published:** 2026-04-13

**Authors:** Victor Perederyi, Eugene Borchik, Viacheslav Zosimov, Oleksandra Bulgakova

**Affiliations:** 1 Admiral Makarov National University of Shipbuilding, Mykolaiv, Ukraine; 2 Mykolayiv National Agrarian University, Mykolaiv, Ukraine; 3 P.M. Platonov Educational and Scientific Institute of Computer Engineering, Automation, Robotics, and Computer Programming, Odesa National University of Technology, Odesa, Ukraine

**Keywords:** Bayesian trust networks, critical infrastructure facilities, expert knowledge, functional stability, fuzzy knowledge base, human factor, hybrid intelligence, information-cognitive technologies

## Abstract

**Introduction:**

Ensuring functional stability of critical infrastructure facilities (CIFs) under conditions of uncertainty and dynamic threats remains a critical challenge. Existing approaches insufficiently integrate technical, cybersecurity, and human-related factors.

**Methods:**

This study proposes an information-cognitive approach based on a hybrid model combining Bayesian Trust Networks and fuzzy logic. The model incorporates expert knowledge and evaluates the mutual influence of information security, cybersecurity, human factors, and vulnerability indicators. The Mamdani algorithm is used for probabilistic estimation under uncertainty.

**Results:**

Numerical experiments conducted in the GeNIe environment demonstrate that the proposed model effectively supports decision-making. Scenario analysis shows that adjusting key cybersecurity and vulnerability factors increases the probability of achieving sufficient functional stability above the critical threshold.

**Discussion:**

The proposed hybrid framework improves interpretability and adaptability of functional stability assessment. It enables flexible reasoning under uncertainty and supports real-time decision-making for critical infrastructure management. The approach can be applied across different categories of CIFs and extended with additional data-driven components.

## 1 Introduction

According to the EU Global Strategy for Foreign and Security Policy, ensuring the resilience of critical infrastructure facilities (CIFs) has become an increasingly important issue. The term “critical infrastructure resilience” is defined by the U.S. Department of Homeland Security (DHS) as the ability to operate reliably under normal conditions, adapt to constantly changing circumstances, withstand threats, and rapidly recover after the realization of threats of any kind. In the broader international research context, this concept is closely related to resilience engineering and sociotechnical system performance, which emphasize the importance of integrating technical, organizational, and human factors when managing safety-critical infrastructure (Hollnagel, 2018, 2025; Woods, 2015).

Currently, there are a significant number of international standards related to information and cybersecurity. For example, ISO/IEC 27000 and ISO/IEC 27004 reflect the current state of information technology and provide recommendations for assessing and managing information security within an organization. However, they do not define specific security requirements for the information security systems that support these infrastructure facilities. Therefore, the security optimization process depends on the purpose and nature of the critical infrastructure facilities (Bagherzadeh et al., 2020; Chio and Freeman, 2018; Kovaliuk et al., 2008).

There are also various standards for methods of assessing the level of information and cybersecurity, such as the Cybersecurity Assessment Standard for Information Technology and the Cyber Security Evaluation Tool (CSET), developed by the U.S. National Institute of Standards and Technology (NIST). These standards allow for assessing cybersecurity within a specific organization and are simpler than other approaches. However, the methodology is time-consuming and complex to implement. The ISO/IEC 27001 standards from the International Organization for Standardization (ISO) are globally recognized and internationally accepted. They are adaptable to different types of organizations and various security standards. Nevertheless, their development and implementation require considerable effort and resources. However, despite their practical importance, existing standards primarily focus on static security assessment procedures and therefore do not provide mechanisms for the dynamic evaluation and adaptation of functional stability in complex critical infrastructure facilities.

Current scientific research in this field primarily addresses the definition of key concepts and reliability indicators, as well as risk analysis and reliability assessment using probabilistic, statistical, and analytical approaches (Lakhno et al., 2022).

In Falcone et al. (2018), two reliability distribution methods are presented, namely the Integrated Factors Method (IFM) and the Critical Flow Method (CFM). However, the study does not provide models or tools for monitoring and supporting the set of indicators for stability and security of complex systems.

The work (Eling and Wirfs, 2019) introduces the threshold exceedance method for identifying “cyber risks and extreme cyber risks.” The main sources of cyber risk factors are identified, which significantly differ from other categories of risks. However, risks related to human behavior are not addressed.

The study (Zosimov et al., 2025) outlines the functional requirements for Smart Grid protection systems. It states that creating an appropriate protection system requires considering new functional protection requirements for future Smart Grids. However, it does not present methods for supporting decision-making processes related to ensuring and monitoring the functional resilience and cybersecurity of complex systems.

In Alali et al. (2018), methods are proposed that allow for the assessment of the overall cybersecurity risk of critical infrastructure facilities (CIFs). The primary sources of information and cybersecurity factors are identified, which differ significantly from other risk categories, such as human behavior. The process of assessing risks arising from erroneous human decisions under the influence of threats is presented. However, the models and methods for evaluating the dependence of CIF functional resilience on threat factors are not explored.

In Cherdantseva et al. (2016), an assessment of the human factor in the cybersecurity of SCADA systems is presented, along with basic protection methods against cyber threats and tools for their implementation. Study (Shin et al., 2017) provides risk assessments of cybersecurity in nuclear facility control systems, proposing a probabilistic approach using Bayesian network models and event trees. Nevertheless, these studies do not propose methods and models for assessing the functional resilience of CIFs based on the state of information and cybersecurity.

General issues related to the development of models and monitoring systems for supporting decision-making in ensuring the functional resilience of CIFs are discussed in Perederyi et al., (2020), Gusenitsa et al. (2020), and Zosimov and Bulgakova (2020). The development of models and IT tools for monitoring and ensuring information and cybersecurity in CIF management is covered in Zheleznov et al. (2020), Hnatushenko and Hnatushenko (2020), and Bulgakova et al. (2021).

Study (Perederyi et al., 2024a, 2024b) explore the construction and investigation of models and methods for identifying and evaluating the influence of security factors on relevant decision-making under uncertainty in CIF management. These are based on information-cognitive technologies. However, they do not address issues of mutual adaptation and adjustment of the human factor, information and cybersecurity status, and functional resilience in accordance with the risk level of the production process and the criticality category of the CIF.

The analysis of existing studies reveals several important limitations. First, many approaches focus primarily on technical or cybersecurity parameters while insufficiently integrating the influence of the human factor on the functional stability of critical infrastructure systems. Second, existing standards and analytical models mainly support static security assessment and provide limited capabilities for dynamic adaptation of functional stability under changing operational conditions. Third, current approaches rarely offer integrated decision-support mechanisms that combine heterogeneous information sources such as expert knowledge, vulnerability indicators, and operational risk factors. These limitations indicate the need for hybrid intelligent methods capable of supporting adaptive and interpretable assessment of functional stability in critical infrastructure facilities.

## 2 Methodology

Based on the requirements of international standards related to information and cybersecurity, as well as the results of the literature review, it has been determined that the most significant factors influencing the functional resilience of CIFs are: information and cybersecurity factors, the vulnerability of CIF information and communication systems, the emotional-cognitive state of users, and the risk level of the production process. These factors were selected as the most influential variables affecting functional stability and were further formalized within the proposed probabilistic model based on expert interpretation of their relationships.

**Figure 1:**
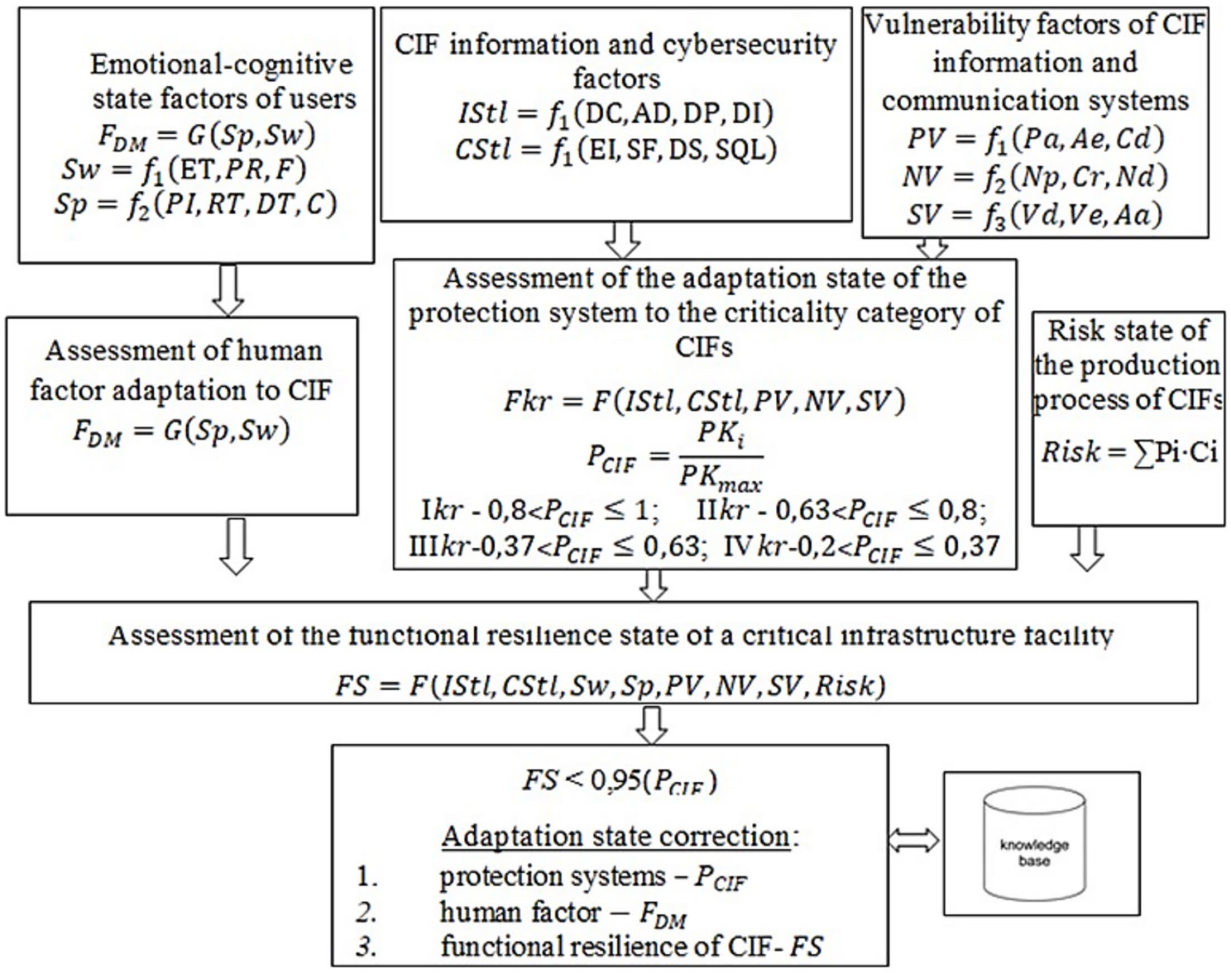
Information-logical model for the assessment and adaptation of the operational state of functional resilience of CIFs.

These factors are illustrated in the information-logical model (Figure 1): factors characterizing psycho-emotional $\mathrm{Sw}=f_{1}(\mathrm{ET},,\mathrm{PR},,F)$ and cognitive state $\mathrm{Sp}=f_{2}(\mathrm{PI},,,\mathrm{RT},,,\mathrm{DT},,,C)$ ; Information security $\text{IStl}=f_{1}(\mathrm{DC},,,\mathrm{AD},,,\mathrm{DP},,,\mathrm{DI})$ ; Cybersecurity $\text{CStl}=f_{1}(\mathrm{EI},,,\mathrm{SF},,,\mathrm{DS},,,\mathrm{SQL})$ ; Vulnerability factors: Physical vulnerabilities $\mathrm{PV}=f_{1}(\mathrm{Pa},,\mathrm{Ae},,\mathrm{Cd})$ , Network vulnerabilities $\mathrm{NV}=f_{2}(\mathrm{Np},,\mathrm{Cr},,\mathrm{Nd})$ , Software vulnerabilities $\mathrm{SV}=f_{3}(\mathrm{Vd},,\mathrm{Ve},,\mathrm{Aa})$ . Where *Sw* is the main factors associated with the current psycho-emotional state of the decision maker (DM), *ET* is an emotional tension, *PR* is a productivity, *F* is a physical and emotional fatigue, *PI* is a perception of information, *RT* is a reaction time, *DT* is a decision-making time, *C* is a concentration, *Pa* – physical attack, *Ae* – access to equipment, *Cd* – copying of data, *Np* –network protocols, *Cr* – access control, *Nd* – network devices, *Vd* – input data validation, *Ve* – vulnerabilities in encryption, *Aa* – Authentication and authorization.

In this study, a Bayesian Trust Network (BTN) is used as the mathematical tool, which has proven effective in modeling complex systems under uncertainty (Perederyi et al., 2024a, 2024b). A BTN is a probabilistic graphical model that represents a set of random variables and their conditional probabilities using a directed acyclic graph. It enables the calculation of the probability values of any node in the network based on the known probabilities of input (root) nodes and the conditional probabilities of the remaining nodes.

**Figure 2:**
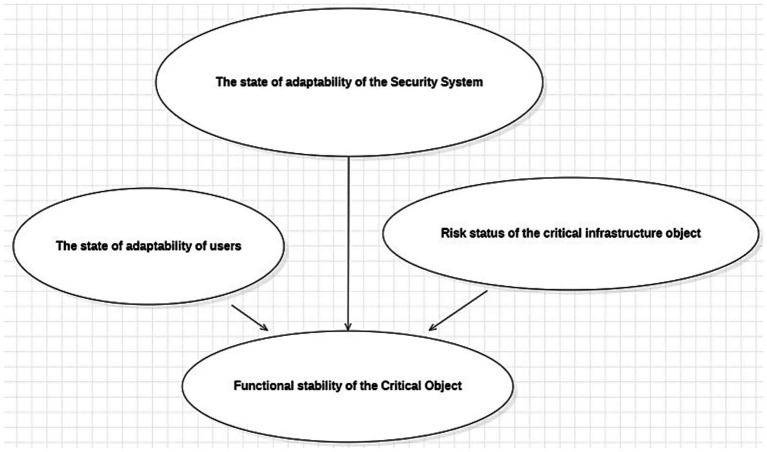
Bayesian trust network for assessing the probability of functional resilience of CIFs.

Based on the information-logical model (Figure 1), this study proposes using a BTN (Figure 2) to assess the degree of functional resilience of critical infrastructure facilities (CIFs). The structure and conditional probability tables of the BTN were developed using expert knowledge. The experts involved in this stage were selected using a formalized pre-panel selection framework described in our previous work (Bulgakova and Zosimov, 2025), in which expert candidates are represented through standardized profile characteristics, including domain competence, professional experience, functional roles, and institutional affiliation, and are admitted under predefined threshold and balance constraints.

To determine the probability values of the root nodes “The state of adaptability of users” and “The state of adaptability of the Security System”, the requirements of engineering and psychological standards for human-operator working conditions (Zosimov and Bulgakova, 2020; Elliott, 2021), as well as the results of expert evaluations, were used. The levels of these factors, expressed in relative units, are presented in Tables 1, 2, Perederyi et al. (2024a), and Perederyi et al. (2024b).

**Table 1:** Significance levels of the emotional and cognitive state factors of the DM.

State	*ЕТ*	*F*	*PR*	*PI*	*RT*	*DT*	*C*
High	0.85–1.0	0.8–1.0	0.75–1.0	0.8–1.0	0.85–1.0	0.9–1.0	0.8–1.0
Medium	0.4–0.85	0.4–0.8	0.45–0.75	0.4–0.8	0.5–0.85	0.5–0.9	0.45–0.8
Low	0.2–0.4	0.25–0.4	0.3–0.45	0.2–0.4	0.2–0.5	0.3–0.5	0.2–0.45

**Table 2:** Significance levels of possible states of vulnerability (IStl) and information and cybersecurity (CStl) factors of CIFs.

Vertex	*Pa*	*Ae*	*Cd*	*Np*	*Cr*	*Nd*	*Vd*	*Ve*	*Aa*	*IStl*	*CStl*
High	0.8–1.0	0.75–1.0	0.85–1.0	0.8–1.0	0.85–1.0	0.8–1.0	0.85–1.0	0.85–1.0	0.85–1.0	0.85–1.0	0.8–1.0
Medium	0.35–0.8	0.45–0.75	0.55–0.85	0.45–0.8	0.4–0.85	0.15–0.8	0.15–0.85	0.4–0.85	0.4–0.85	0.4–0.85	0.25–0.8
Low	0.2–0.35	0.25–0.45	0.3–0.55	0.15–0.45	0–0.4	0–0.15	0–0.15	0–0.4	0.15–0.4	0–0.4	0–0.25

All network nodes are binary, meaning they have two states, and with the exception of the node “Functional stability of the Critical Object”, they are of the “Chance – General” type. For example, the vertex “Risk status of the critical infrastructure object” takes the value “occurs” if the risk of disruption to the CIF’s production process exists, and “not occurs” otherwise.

If the adaptation state of the human factor to the CIF is acceptable, the vertex “The state of adaptability of users” takes the value “sufficient”, and “not sufficient” if it is not. Similarly, the vertex “The state of adaptability of the Security System” takes the value “sufficient” if the system’s adaptation to the CIF is deemed acceptable, and “not sufficient” otherwise.

The random variable “The state of adaptability of the Security System” is influenced by information and cybersecurity factors of the CIF (*IStl* and *CStl*) (see Figure 1), as well as vulnerability factors of the CIF’s information and communication (*IC*) systems – namely: *Pa*, *Ae*, *Cd*, *Np*, *Cr*, *Nd*, *Vd*, *Ve*, *Aa*. To estimate the probability that the variable “The state of adaptability of the Security System” takes the value “sufficient” (or “not sufficient”), a probability forecasting system is proposed, based on fuzzy inference using the Mamdani algorithm and a fuzzy knowledge base [33, 34], in which the input and output variables are defined by fuzzy sets.

Considering that, according to Perederyi et al. (2024a) and Perederyi et al. (2024b), the most significant influence on the adaptability of the CIF’s protection system comes from the factors *Np*, *Aa*, *IStl*, *Cd*, *Ve*, the following fuzzy knowledge base has been proposed by experts:

RULE 1: IF *u*_1_ is “high” AND *u*_2_ is “high” AND *u*_3_ is “medium” AND *u*_6_ is “medium” THEN *v is* “low.”

RULE 2: IF *u*_2_ is “high” AND *u*_3_ is “low” AND *u*_8_ is “low” AND *u*_6_ is “medium” THEN *v is* “low.”

RULE 3: IF *u*_3_ is “low” AND *u*_5_ is “medium” AND *u*_6_ is “medium” AND *u*_7_ is “medium” THEN *v is* “low.”

RULE 4: IF *u*_4_ is “medium” AND *u*_3_ is “low” AND *u*_5_ is “medium” AND *u*_6_ is “medium” AND *u*_7_ is “medium” THEN *v is* “low.”

RULE 5: IF *u*_1_ is “low” AND *u*_3_ is “high” AND *u*_4_ is “medium” AND *u*_5_ is “low” AND *u*_6_ is “medium” THEN *v is* “medium.”

RULE 6: IF *u*_2_ is “low” AND *u*_3_ is “medium” AND *u*_4_ is “low” AND *u*_5_ is “medium” AND *u*_6_ is “high” AND *u*_7_ is “medium” THEN *v is* “medium.”

RULE 7: IF *u*_2_ is “low” AND *u*_3_ is “high” AND *u*_4_ is “medium” AND *u*_5_ is “high” AND *u*_6_ is “high” AND *u*_7_ is “medium” AND *u*_8_ is “low” AND *u*_9_ is “medium” THEN *v is* “medium.”

RULE 8: IF *u*_6_ is “medium” AND *u*_4_ is “low” AND *u*_1_ is “low” AND *u*_2_ is “medium” AND *u*_3_ is “high” AND *u*_7_ is “medium” THEN *v is* “medium.”

RULE 9: IF *u*_1_ is “low” AND *u*_2_ is “low” AND *u*_3_ is “high” AND *u*_4_ is “medium” AND *u*_5_ is “low” AND *u*_6_ is “high” AND *u*_7_ is “low” AND *u*_9_ is “medium” AND *u*_10_ is “low” THEN *v is* “high.”

RULE 10: IF *u*_6_ is “high” AND *u*_4_ is “low” AND *u*_1_ is “low” AND *u*_2_ is “low” AND *u*_3_ is “high” AND *u*_7_ is “low” AND *u*_10_ is “medium” AND *u*_11_ is “medium” THEN *v is* “high.”

RULE 11: IF *u*_1_ is “low” AND *u*_2_ is “low” AND *u*_3_ is “high” AND *u*_4_ is “low” AND *u*_5_ is “low” AND *u*_6_ is “high” AND *u*_7_ is “low” AND *u*_8_ is “low” AND *u*_9_ is “low” AND *u*_10_ is “high” AND *u*_11_ is “high” THEN *v is* “high.”

Where *u*_i_ (*i* = 1, … 11) are the linguistic variables: *u*_1_ is level of usage of unprotected *Np*, *u*_2_ is level of vulnerability in *Aa*, *u*_3_ is level of *IStl*, *u*_4_ is level of *Cd*, *u*_5_ is level in *Ve*, *u*_6_ is level of *CStl*, *u*_7_ is level of *Vd* vulnerabilities, *u*_8_ is level of *Cr*, *u*_9_ is level of vulnerabilities of *Nd*, *u*_10_ is level of *Ae*, *u*_10_ is level of protection against remote *Pa*, *v* – linguistic variable denoting the probability that the random variable “The state of adaptability of the Security System” assumes the value “sufficient,” as defined within the fuzzy inference system, {“low,” “average,” “high”} is a term-set of the variables *u*, *v*.

The fuzzy rule base was constructed to ensure coverage of the main combinations of input factors identified in the information-logical model. The rules represent typical system states corresponding to different levels of user adaptability and security system adaptability. The resulting 11 rules correspond to the main operational configurations of these factors and provide sufficient coverage of the resilience assessment scenarios considered in the study.

The mathematical formulation of the proposed model is presented in Equations (1-5). The membership function $\mu_{1i}(x_{i})$ of the term “low” of linguistic variables $u_{i}(i=\bar{5,8};i=10;11)$ , $\mu_{2i}(x_{i})$ , of the term “medium” of linguistic variables $u_{i}(i=\bar{1,11})$ will be given in the form of a symmetric Gaussian function:

$$\mu_{\mathrm{mi}}(x_{i})=\text{gauss}\mathrm{fm}(x_{i},[\sigma_{\mathrm{mi}},c_{\mathrm{mi}}])=e^{\frac{-{\left(x_{i}-c_{\mathrm{mi}}\right)}^{2}}{2\sigma_{\mathrm{mi}}^{2}}}$$ (1)

Where parameters $\sigma_{\mathrm{mi}}>0;c_{\mathrm{mi}}\geq 0,(m=\bar{1,3})$ , *x_i_* are an elements of the universal set *X*, on which the terms of linguistic variables are defined.

The membership function $\mu_{1i}(x_{i})$ of the term “low” of linguistic variables $u_{i}(i=\bar{1,4};i=9)$ will be given in the form of a two-sided Gaussian function:

$$\begin{array}{c} \mu_{i}(x_{i})=\text{gauss}2\mathrm{fm}(x_{i},[\sigma_{1i}^{1},c_{1i}^{1},\sigma_{1i}^{2},c_{1i}^{2}])=\\ =\{\begin{array}{c} e^{-{\left(x_{i}-c_{1i}^{1}\right)}^{2}/2{\left(\sigma_{1i}^{1}\right)}^{2}},\text{if}\;0\leq x_{i}\leq c\\ 1,\text{if}\;c_{1i}^{1}<x_{i}<c_{1i}^{i}\\ e^{-{\left(x_{i}-c_{1i}^{1}\right)}^{2}/2{\left(\sigma_{1i}^{1}\right)}^{2}},\text{if}\;x_{i}\geq c_{1i}^{2} \end{array} \end{array}$$ (2)

Where $\sigma_{1i}^{1},\sigma_{1i}^{2}>0;c_{1i}^{1},c_{1i}^{2}\geq 0;c_{1i}^{1}<c_{1i}^{2}(i=\bar{1,4})$ .

Taking into account the significance levels of possible states of the vulnerability and information and cybersecurity factors of CIFs (Table 2) makes it possible to determine the parameters $c_{\mathrm{mi}},c_{1i}^{1},c_{1i}^{2}$ of the Gaussian functions (1), (2): $c_{1i}=0\;(i=\bar{5,8};i=10;11)\;$ ; $\begin{array}{l} c_{21}=0.575;c_{22}=0.6;c_{23}=0.7;c_{26}=0.47;c_{27}=0.5;c_{2\;11}=0.525;c_{24}\\ =c_{25}=c_{28}=c_{29}=c_{2\;10}=0.625;c_{3i}=1\;(i=\bar{1,11});c_{1i}^{1}=0\;(i=\bar{1,4};i=9)\\ ;c_{11}^{2}=0.2;c_{12}^{2}=0.25;c_{13}^{2}=0.3;c_{14}^{2}=0.15;c_{19}^{2}=0.15. \end{array}$

Since $0\leq x_{i}\leq 1$ , it follows from the condition $c_{1i}^{1}=0\;(i=\bar{1,4};i=9)$ that the corresponding parameters of the two-sided Gaussian function $\sigma_{1i}^{1}$ can take any positive values. It is further assumed that $\sigma_{11}^{1}=\sigma_{12}^{1}=\sigma_{14}^{1}=\sigma_{19}^{1}=1$ . For the membership functions of the terms “low,” “medium,” and “high” of the linguistic variable *v*, the following notations $\psi_{i}(y),i=\bar{1,3}$ are introduced, where y∈Y={0≤y≤1} , the universal set over which these terms are defined. The function $\psi_{i}(y)$ is defined as a symmetric Gaussian function:

$$\psi_{i}(y)=\text{gauss}\mathrm{fm}(y,[\sigma_{i},c_{i}])=e^{\frac{-{\left(y-c_{i}\right)}^{2}}{2\sigma_{i}^{2}}}$$ (3)

**Figure 3:**
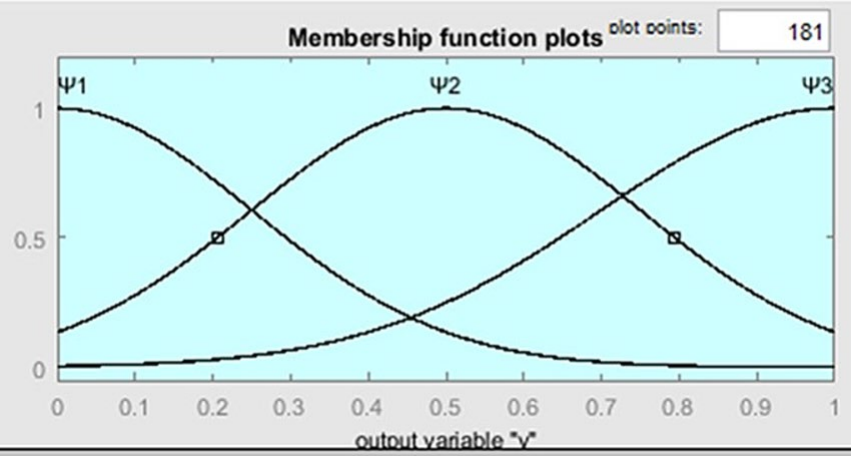
The graph of membership functions $\psi_{i}(y),i=\bar{1,3}$ .

**Figure 4:**
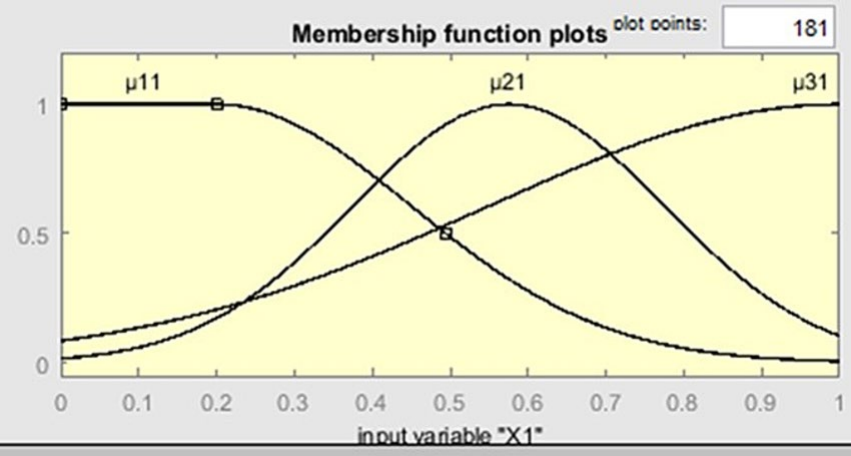
The graph of membership functions μ_11_(*x*1), μ_21_(*x*1), μ_31_(*x*1).

**Figure 5:**
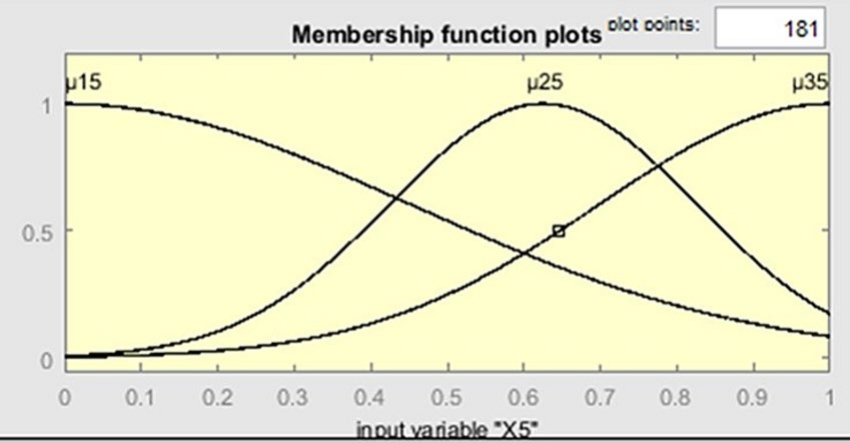
The graph of membership functions μ_15_(*x*_5_), μ_25_(*x*_5_), μ_35_(*x*_5_).

Where parameters $\sigma_{i}>0;c_{i}>0,i=\bar{1,3}$ . Below are the characteristic graphs of the membership functions (Figure 3). The graphs of the functions $\mu_{12}(x_{2}),\mu_{13}(x_{3}),\mu_{14}(x_{4}),\mu_{19}(x_{9})$ have the same shape as the graph of the function $\mu_{11}(x_{1})$ (Figure 4). The graphs of the functions $\mu_{16}(x_{6}),\mu_{17}(x_{7}),\mu_{18}(x_{8}),\mu_{1\;10}(x_{10}),\mu_{1\;11}(x_{11})$ have the same shape as the graph of the function $\mu_{1\;15}(x_{5})$ (Figure 5).

For configuring the fuzzy model *F*, i.e., to determine the coefficients of the model $\begin{array}{l} \sigma_{11}^{2},\sigma_{12}^{2},\sigma_{13}^{2},\sigma_{14}^{2},\sigma_{15},\sigma_{16},\sigma_{17},\sigma_{18},\sigma_{19}^{2},\sigma_{1\;10},\sigma_{1\;11},\sigma_{2i},\sigma_{3i},\sigma_{j},c_{j};\\ i=\bar{1,11};j=\bar{1,3}, \end{array}$ it is required that the value of root mean square deviation should be minimized:

$$R=\frac{1}{n}\sum_{k=1}^{n}{\left(y_{k}-F(P,E_{k})\right)}^{2}\to \min$$ (4)

Where *n* is the volume of the sample of experimental data connecting the inputs *E* = (*x*_1_, *x*_2_, *x*_3_, *x*_4_, *x*_5_, *x*_6_, *x*_7_) to the output *y* of the investigated dependence:

$$(E_{k}-y_{k}),k=\bar{1,n}$$ (5)

Where *E_k_* = (*x*_*k,*1_, *x*_*k,*2_, *x*_*k,*3_, *x*_*k,*4_, *x*_*k,*5_, *x*_*k,*6_, *x*_*k,*7_) is a vector of inputs and *y_k_* is an output in *k*-pair. In addition, *F*(*P*, *E_k_*) is the value of the output of the fuzzy model at the value of the inputs specified by the vector *E_k_*; $P=(\sigma_{1j}^{2},\sigma_{\mathrm{mi}},\sigma_{l},c_{l})$ is a vector of coefficients of membership functions of terms of input and output variables of the fuzzy model. The experimental dataset used in the optimization procedure was generated from expert assessments of representative combinations of the input factors related to vulnerability, information security, and cybersecurity conditions of the critical infrastructure facility.

Taking into account expert knowledge on the influence of vulnerability factors of CIF information and communication systems, as well as information and cybersecurity factors, on the degree of adaptability of the protection system allows for solving a mathematical programming problem using the Fuzzy Logic Toolbox and Optimization Toolbox, and thus configuring the fuzzy model.

A similar approach is used to estimate the probability that the random variable “The state of adaptability of users” takes the value “sufficient.”

To describe the node “Functional stability of the Critical Object,” which can take the values “sufficient” and “not sufficient,” experts were asked to assess the conditional probabilities of its possible states. The results are presented in Table 3. This node is assigned the Noisy MAX type. In this case, the conditional probabilities are determined by the independent influence of the contributing factors on the expected event rather than their joint influence, which simplifies the task of estimating conditional probabilities for experts.

**Table 3:** Conditional probabilities of the vertex “Functional stability of the Critical Object”.

Parent	The adaptability state of users		The adaptability state of the security system		Risk state of the critical infrastructure facility	
State	Not sufficient	Sufficient	Not sufficient	Sufficient	Not sufficient	Sufficient
Not sufficient	0.2	0	0.25	0	0.35	0
Sufficient	0.8	1	0.75	1	0.65	1

## 3 Results

To test the proposed information technology, the following numerical calculation was carried out for the functional stability (FS) of a critical infrastructure facility (CIF) of the first criticality category, with a critical probability value of *P_cr_* = 0.95 (also denoted as *P_CIF_*). Two scenarios were considered.

In the first scenario, based on the results of decision-maker testing and the indicators from the hardware and software sensors of the protection system, the following values of the influencing parameters related to information and cybersecurity and the vulnerability of the CIF’s information and communication systems were recorded (see Tables 4, 5).

**Table 4:** Values of the emotional-cognitive state factors of the DM.

Level	*ЕТ*	*F*	*PR*	*PI*	*RT*	*DT*	*C*
High	–	–	0.8	0.9	–	–	0.9
Medium	0.5	0.6	–	–	–	–	–
Low	–	–	–	–	0.3	0.4	–

**Table 5:** Recorded values of the CIF’s vulnerability and information and cybersecurity factors (Scenario 1).

Level	*Pa*	*Ae*	*Cd*	*Np*	*Cr*	*Nd*	*Vd*	*Ve*	*Aa*	*IStl*	*CStl*
High	0.9	–	–	–	–	–	–	–	–	–	–
Medium	–	0.5	–	–	–	–	–	0.6	–	–	0.4
Low	–	–	0.4	0.2	0.1	0.1	0.1	–	0.35	0.3	–

Based on the probability forecasting system proposed in this study, which is grounded in fuzzy inference using the Mamdani algorithm and a fuzzy knowledge base, the probabilities of the nodes in BTN: “The state of adaptability of users” and “The state of adaptability of the Security System” were calculated in MATLAB.

As a result, the probability that the users’ adaptability state is sufficient was found to be 0.98, while the probability that the adaptability of the CIF vulnerability-related factors is sufficient was 0.8.

It was assumed that the probability of a production process risk state is low and equals 0.01. The final calculation, performed in GeNIe 4.1 using the BTN and the specified unconditional and conditional probabilities, showed that the probability of the CIF being in a state of functional stability was: *P*(FS = sufficient) = 0.94. Although the difference between the obtained probability value and the critical threshold is numerically small, it has practical significance in the context of risk management. In the proposed framework, the threshold *P_cr_* represents the boundary between acceptable and critical levels of functional stability; therefore, even a deviation of 0.01 may affect the classification of the system state and indicate the need for additional monitoring or corrective actions.

Since this probability is lower than the critical threshold *P_cr_* = 0.95, the CIF cannot be considered functionally stable under the given conditions.

In the second scenario, the emotional and cognitive state factors of the DM, as well as the probability of the production process risk state, remain the same as in the first scenario.

As shown in Figure 5, the probability of a sufficient level of security system adaptability is 80%. This indicates that by adjusting the values of the influencing factors related to information and cybersecurity, the probability of achieving a sufficient level of adaptability of the security system can be significantly increased.

This, in turn, should lead to an improvement in the functional resilience of the CIF.

As seen in Table 5, the factors representing the IStl and CStl take on low (0.3) and medium (0.4) values, respectively. To increase the functional resilience of the CIF, the values of these factors must be raised.

The modified factor values are presented (in bold) in Table 6.

**Table 6:** Recorded values of the CIF’s vulnerability and information and cybersecurity factors (Scenario 1).

Level	*Vd*	*Ve*	*Aa*	*IStl*	*CStl*
High	–	–	–	**0.9**	**0.85**
Medium	–	0.6	–	–	–
Low	0.1	–	0.35	–	–

Bold values indicate modified factor values compared to Scenario 1.

The probability that the adaptability of the security system is sufficient is estimated using the Mamdani algorithm based on the values of the aforementioned factors (Table 6), and equals 0.97.

The calculation performed on the BTN in the GeNIe 4.1 environment, using the specified unconditional and conditional probabilities, shows that the probability of the CIF being in a state of functional stability in this scenario is *P*(FS = sufficient) = 0.97.

Since this probability exceeds the critical threshold *P_cr_* = 0.95, the CIF can be considered functionally stable within its assigned criticality category.

The obtained results of the numerical experiments are consistent with real-world decision-making practices regarding the assurance of functional stability of CIFs according to their criticality category.

In the first scenario, the underestimated values of information and cybersecurity protection factors had a negative impact on the functional stability of the CIF. In this case, adjustment of these factors is required in accordance with ISO/IEC 27001 standards. In the second scenario, after adjusting these factors and keeping the other values unchanged, the probability of a sufficient level of functional stability increased to the required level.

## 4 Discussion

This study addresses the problem of assessing and adapting the functional stability (FS) of critical infrastructure facilities (CIFs) under conditions of uncertainty, limited data availability, and heterogeneous influencing factors. Unlike traditional approaches that consider cybersecurity, reliability, or human factors in isolation, the proposed information-cognitive technology integrates security-related parameters, vulnerability aspects, the human factor, and production-process risks within a unified probabilistic framework. This integration reflects the systemic nature of CIF operation and aligns with the concept of functional stability as an adaptive, rather than purely static, property.

A key methodological contribution lies in the hybrid use of Bayesian Trust Networks (BTN) and fuzzy inference. The BTN enables explicit causal modeling of dependencies between factors influencing FS, while fuzzy logic supports the estimation of probabilistic inputs when quantitative measurements are incomplete or unavailable. This combination is particularly relevant for CIF environments, where statistical datasets are often restricted for security reasons and expert knowledge plays a decisive role. The use of linguistic variables and membership functions allows expert judgments to be formalized without forcing unrealistic numerical precision.

The scenario-based experiments demonstrate the practical applicability of the proposed approach for decision support. In the first scenario, the probability of achieving sufficient functional stability does not reach the required threshold for a first-category CIF, indicating an unacceptable operational state. In the second scenario, targeted improvements in information security and cybersecurity parameters lead to a measurable increase in the probability of sufficient FS, exceeding the critical threshold. Importantly, this improvement is achieved without modifying assumptions related to the human factor or production risks, highlighting the value of focused adaptive interventions. Such a result is consistent with real-world CIF management practices, where corrective actions are constrained by time, resources, and regulatory requirements.

From an interpretability perspective, the BTN structure and the use of Noisy-MAX nodes reduce the complexity of probability elicitation and facilitate understanding by decision makers. This is a significant advantage over black-box predictive models, especially in safety-critical and regulated domains where transparency and justification of decisions are mandatory. The model also supports exploratory “what-if” analyses, enabling stakeholders to assess the expected impact of specific control measures on overall functional stability before their implementation.

At the same time, several limitations should be noted. The presented results are based on expert-defined network structures, fuzzy rules, and membership functions, which introduces subjectivity and potential bias. The current binary representation of node states simplifies interpretation but may not fully capture intermediate or degraded operational modes. Additionally, the study focuses on static scenarios, whereas real CIF systems operate in a dynamic environment with evolving threats, operator conditions, and system states.

These limitations point to directions for further research. Future work may involve extending the model to multi-state or continuous representations, incorporating learning mechanisms for conditional probabilities based on historical or monitoring data, and performing sensitivity analyses to identify the most influential factors across different CIF categories. Validation on a broader set of infrastructure types and integration with real-time monitoring and incident response systems would further enhance the practical relevance of the approach.

## 5 Conclusion

This paper presents an information-cognitive approach to assessing and adapting the functional stability of critical infrastructure facilities under uncertainty. Functional stability is considered as an integrated and adaptive property that depends on cybersecurity and information security measures, vulnerability-related factors, the human factor, and the risk state of production processes. By unifying these heterogeneous influences within a single probabilistic framework, the proposed approach supports consistent and transparent decision making in safety-critical infrastructure contexts.

The combination of Bayesian Trust Networks with fuzzy inference enables the use of expert knowledge when precise statistical data are unavailable, while preserving causal interpretability and the ability to perform scenario-based analysis. The demonstrated case scenarios show that functional stability can be improved through targeted adjustments of controllable security parameters, allowing the probability of a sufficient stability state to exceed critical thresholds defined for high-importance infrastructure categories. This confirms the practical value of the approach for evaluating and justifying adaptive security measures. The general structure of the proposed model allows it to be adapted to different categories of critical infrastructure facilities by adjusting the set of input factors, threshold parameters, and expert knowledge used in the probabilistic and fuzzy components. This flexibility also enables the framework to be applied under different national regulatory environments where specific cybersecurity standards and risk criteria may vary.

The proposed framework is particularly suitable for CIF management environments that require explainable assessments and risk-informed adaptation strategies. In practice, the proposed Bayesian-fuzzy model can be integrated into real-time monitoring or decision-support platforms used in critical infrastructure management, where it may assist operators in continuously evaluating functional stability and identifying situations requiring preventive or corrective actions. Future work will focus on extending the model to dynamic and multi-state representations, incorporating learning mechanisms based on operational data, and validating the approach on a wider range of critical infrastructure types and real-world operating conditions.

## Data Availability

The raw data supporting the conclusions of this article will be made available by the authors, without undue reservation.
